# Evaluation of Workability and Crack Resistance of Recycled Plastic Asphalt Mixtures

**DOI:** 10.3390/polym17212840

**Published:** 2025-10-24

**Authors:** Haosen Jing, Riccardo Monticelli, Claudia Graiff, Laura Bergamonti, Elena Romeo, Gabriele Tebaldi

**Affiliations:** 1 Department of Engineering and Architecture, University of Parma, Parco Area delle Scienze 181/A, 43124 Parma, Italy; riccardo.monticelli@unipr.it; 2 Department Chemistry, Life Sciences and Environmental Sustainability, University of Parma, Parco Area delle Scienze 17/A, 43124 Parma, Italy; claudia.graiff@unipr.it (C.G.); laura.bergamonti@unipr.it (L.B.)

**Keywords:** asphalt mixture, plastic, food package, Superpave IDT, crack

## Abstract

To address the global plastic crisis, recycled plastics from food packaging were used as road materials by the dry method for practical application research. First, the main components of the recycled plastics were identified based on FTIR, and their thermal stability was evaluated through DSC, TG, and microscopic analysis. Then, the workability of the plastic–asphalt mixture was evaluated using the gyratory compaction indicator, void content, and compaction energy index (CEI). Finally, the effect of reused plastics on the cracking resistance of bituminous mixtures was examined with the Superpave IDT test. The results indicate that recycled plastics from food packaging are polyolefin composite materials, primarily consisting of Low-Density Polyethylene (LDPE), Linear Low-Density Polyethylene (LLDPE), High-Density Polyethylene (HDPE), and Polypropylene (PP), and that their thermal stability meets production requirements. Good compaction performance was observed with plastic content below 2% of the aggregate weight, while higher contents reduced void content due to the space occupied by plastics. When the plastic content increased from 0.5% to 2.0%, creep compliance decreased from 68.4% to 77.87%, while the m-value, tensile strength, and elastic energy maximum decreased by 30.77%, 5.6%, and 7%, respectively. In contrast, the failure strain, fracture energy, and maximum DSCE increased by 25.86%, 87.43%, and 133.05%, respectively. The recycled plastic enhanced the toughness of the asphalt mixture, increasing the dissipated energy during crack propagation and improving its resistance to permanent deformation. Moreover, the plastics hindered crack propagation through a bridging effect, leading to fewer cracks within plastic zones compared with surrounding areas. This study provides actionable guidance for the application of composite plastics in asphalt pavements and supports their sustainable development.

## 1. Introduction

Due to the widespread use of plastics in daily life, plastic production exceeded 400 million tons in 2023, and global plastic production is projected to attain 1.1 billion tons by 2050 [1,2,3]. Most plastics are improperly disposed of, polluting the environment. OECD data show that the global recycling rate for plastic waste is merely 9% [4]. Large amounts of plastic are burned or landfilled, spreading through the air and water into the ocean and soil [5]. Plastics have a long half-life in the natural environment and are difficult to degrade. Due to their ubiquity and resistance to degradation, plastic pollution has become a serious and irreversible global environmental challenge [6]. Therefore, research on the application of recycled plastics is becoming increasingly important.

The environmental accumulation of plastics can be reduced by using recycled plastics in road construction on a large scale, which is considered an effective approach. Given their characteristics, most plastics are used for disposable packaging and are discarded as waste within one year of manufacture [7]. Common waste plastics include polyethylene (PE) and polypropylene (PP) from food packaging, and polyethylene terephthalate (PET) from plastic bottles [8]. At present, plastic recycling is mainly divided into mechanical and chemical recycling. Mechanical recycling involves reprocessing the plastic into uniformly sized plastic particles without significantly changing its chemical structure [9]. The essence of chemical recycling is the reconstruction and transformation of chemical structures. Taking thermal depolymerization as an example, which is the most mature technology in chemical recycling, it breaks the C–C bonds by heating to form smaller organic molecules from long polymer chains [10]. Because of their prevalence, mechanically recycled plastics have garnered increasing research attention.

There are two main methods for applying plastics to asphalt pavement construction: the dry method and the wet method. In the wet method, plastic is mixed with asphalt as a modifier and enters the road life cycle in the form of modified asphalt [11]. In the dry method, plastics are used as aggregate substitutes or are directly added to the mixture. They are first mixed with preheated aggregates and then combined with other materials [12]. The dry method is divided into two forms based on the melting point. One is that high–melting-point plastic directly replaces fine aggregates, and the other is that low–melting-point plastic coats the aggregates to form a protective film, replacing part of the asphalt [13,14]. The main problem faced by the wet process is poor compatibility, which requires the use of grafting agents such as maleic anhydride to improve compatibility [15]. Compared with the wet process, the dry method does not require modification of existing equipment and offers a higher utilization rate and greater economic benefits.

At present, the application of plastics in road engineering has gradually become a research hotspot. PE is the most widely studied plastic because 50% of waste plastics come from food packaging, which mainly uses PE [16]. For wet method, research shows that asphalt containing 5% PE has higher rutting resistance and a longer fatigue life than ordinary asphalt [17]. The rutting performance of modified asphalt containing 3% HDPE is comparable to that of bitumen modified by SBS. The water stability of the asphalt mixture is improved, and its overall performance meets the requirements of high-traffic roads [18]. For the dry process, adding 1.5% of PE by weight of the mixture can enhance resistance to permanent deformation and cracking of the asphalt mixture without any negative effects [19]. Even when PE is mixed at temperatures higher than its melting point, research shows that it does not completely melt but forms elastic particles, and the compaction degree is higher than that of ordinary asphalt mixtures [20]. Current research mainly focuses on single-component plastics, and the impact of composite plastics on the performance of asphalt mixtures still needs further evaluation.

Cracking is the primary distress that affects the durability of roads. Once cracking occurs, pavement deteriorates rapidly due to water erosion and freeze–thaw cycles [21]. The cracking performance of plastic–asphalt mixtures has always been a focus of research and has received widespread attention [22,23]. There is currently debate over the impact of plastics on asphalt mixture cracking. Some researchers believe that plastics act as a bridge to slow the cracking process [24,25,26], while others argue that plastics accelerate cracking [27]. The latest research suggests that the effect of plastic on cracking depends on dosage, and that too high a dosage accelerates crack propagation [28,29]. However, these theories and results mainly concern single-component plastics, and it remains unclear whether they are applicable to composite plastics from food packaging.

## 2. Scope and Objectives

This work aims to study the application workability and crack resistance of recycled plastics from food packaging at different dosages. First, the main components of the recycled plastics were analyzed using FTIR, and the thermal stability of the plastics was studied in combination with DSC, TG and microscopic images. Then, the workability of the plastic–asphalt mixture was evaluated using the compaction index, void content and compaction energy index (CEI). Finally, the energy threshold was calculated by the Superpave IDT to evaluate the fracture resistance of the plastic-reinforced asphalt mixture. This work supports the application of recycled plastics from food packaging in actual road construction, improving the sustainability of pavements.

## 3. Raw Materials and Methods

### 3.1. Raw Materials

**Figure 1:**
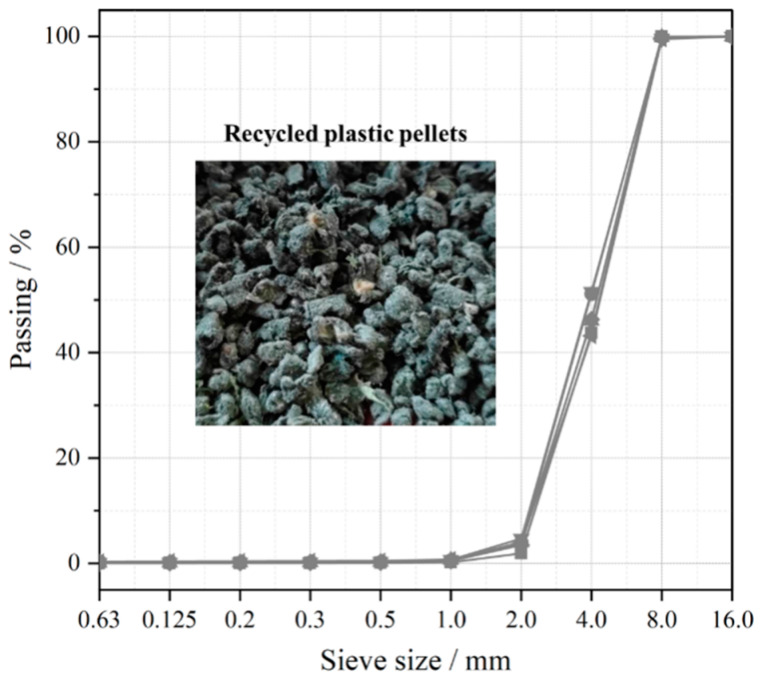
Plastic pellet distribution.

Recycled plastics originate from food packaging and have a complex composition due to their multi-layered structure [30]. Food packaging plastics were processed by a professional plastic recycling company through compression, grinding, and melting, and then turned into uniform recycled plastics. The plastic particles were irregular in shape, less than 10 mm in size, and had a melt flow rate of 4.0 g–4.5 g/10 min at 190 °C/2.16 kg. Figure 1 shows the size distribution of the recycled plastic particles. The consistent results after 6 repeated screenings confirm the reliability of the material.

### 3.2. Methods

#### 3.2.1. Plastic-Modified Asphalt Mixture Preparation

**Figure 2:**
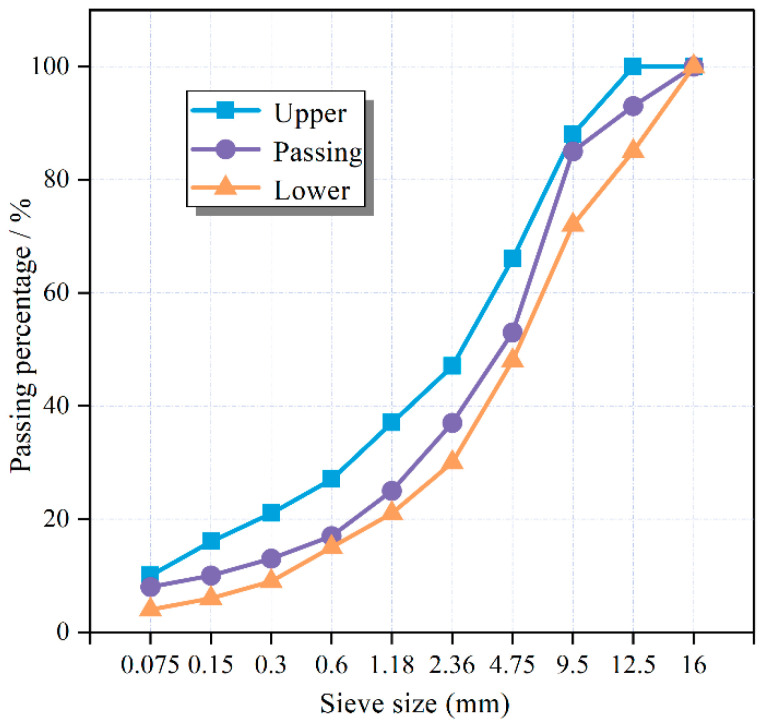
Plastic asphalt pavement gradation curve.

The mixture was prepared using asphalt with a penetration grade of 50–70 and a PG rating of 58–22. The grading curve used in this work is shown in Figure 2. Plastics at 0–2% of the aggregate weight were added to asphalt mixtures based on the dry process: (1) Preheat the aggregate in an oven at 170 °C for 4 h, then mix it with the cold plastic at 165 °C for 5 min to distribute the plastic evenly; (2) Add 5.2% asphalt by weight of aggregate and mix for 5 min; (3) Add filler and mix for an additional 5 min. The aggregate mass of each specimen was 4.5 kg, and two indirect tensile specimens were obtained by cutting.

#### 3.2.2. FTIR Test

The properties of recycled packaging plastics are determined by their composition, and FTIR serves as a widely employed analytical technique for characterizing the functional groups of materials. PerkinElmer FTIR spectroscopy was used to determine the chemical composition of the plastics, employing ATR mode with a diamond crystal. The scanning range was set between 400 cm^−1^ and 4000 cm^−1^.

#### 3.2.3. Thermal Stability Evaluation

As recycled plastics replace conventional materials, their thermal stability during the production process must be determined [31]. The thermal stability of the recycled plastics was evaluated using a PerkinElmer DSC 6000 (PerkinElmer, Waltham, MA, USA), covering an actual production temperature range of 10–300 °C at a heating rate of 10 °C/min.

Thermogravimetric analysis (TGA) of the recycled plastics was performed using a DTG-60H type instrument with an airflow rate of 40 mL/min and heating temperatures ranging from 20 °C to 800 °C.

To simulate the mixing and transportation process, the plastic particles were subjected to thermal treatment in an oven at 180 °C for a duration of 6 h. The morphology of the thermally treated plastics was observed using an optical microscope to evaluate potential structural changes.

#### 3.2.4. Workability Evaluation

The compaction degree and void content of asphalt mixtures are affected by plastics, which influence practical engineering performance [32]. The compaction density was determined based on the specimen height during the gyratory compaction process, and the compaction index was calculated according to Equation (1).

(1) $$\mathrm{Compaction}\;\mathrm{index}=\frac{G_{mb,N}}{G_{mm}}=\frac{4m}{\pi D^{2}h\times G_{mm}}$$

where *G_mb,N_* is the density at the Nth compaction; *G_mm_* is the maximum theoretical density; *m* is the mass of the asphalt mixture; *D* is the diameter of the specimen; and *h* is the height of the specimen at the Nth rotation.

According to AASHTO T166 and AASHTO T209, the bulk density (*G_mb_*) and maximum theoretical density (*G_mm_*) of the samples were measured, respectively [33]. The void content was calculated according to Equation (2).

(2) $$\mathrm{Void}\;\mathrm{content}=(1-G_{mb}\times {G_{mm}}^{-1})\times 100\%$$

**Figure 3:**
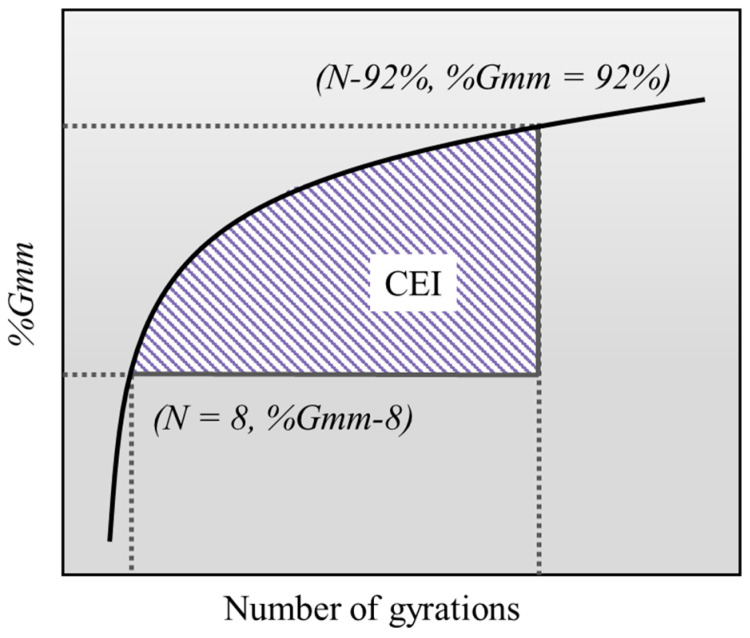
Compaction energy index.

To further characterize the workability of the plastic–asphalt mixture (Figure 3), compaction during the construction phase was evaluated using the compaction energy index (CEI). The CEI represents the calculated compaction energy of the mixture from *N* = 8 to *%G_mm_* = 92% of the compaction curve, indicating the compactability of the asphalt mixture during construction [34].

#### 3.2.5. Optical Microscope

A Zeiss Axiocam 208 color optical microscope was used to observe the target material characteristics. It has a pixel resolution of 3840 (horizontal) × 2160 (vertical) = 8.3 megapixels, a maximum rate of 30 frames per second, and a spectral sensitivity range of 400–700 nm. Micron-level structures can be easily identified with 4K UHD resolution and objective lens magnification. The main observation targets in this work were (1) the high temperature melting of plastics (heating conditions as described in Section 3.2.3); (2) the morphology of plastics in the mixture (observed from IDT samples); and (3) the influence of plastics on the cracking process (cross-sections of specimens after the IDT fracture test).

#### 3.2.6. Superpave IDT Test

The Superpave IDT serves as a key procedure for determining the crack resistance of asphalt mixtures, calculating the damage energy threshold as defined by the HMA-FM model [35]. Compared with Paris’ law, the HMA-FM model considers microcrack healing effects [36]. Fracture occurs only when the accumulated damage during loading exceeds the healing rate [37]. Therefore, the Superpave IDT is an effective method for assessing the crack resistance of plastic-modified asphalt mixtures.

**Figure 4:**
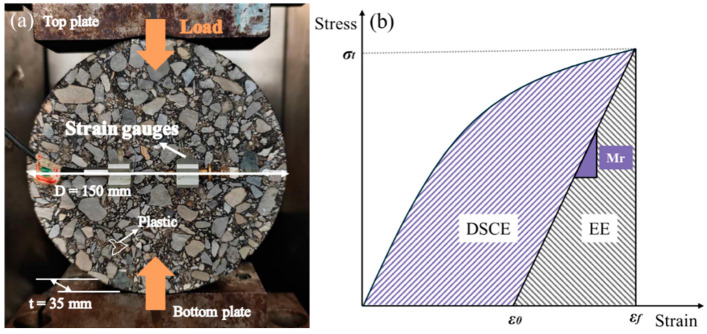
IDT test: (**a**) specimen placement; (**b**) fracture energy diagram.

During testing, a cylindrical sample was subjected to a compressive load to produce a uniform tensile stress perpendicular to its diameter [38]. As shown in Figure 4a, cylindrical samples measuring 150 mm in diameter and 35 mm in height were mounted and secured on the MTS equipment platform and fixed by a mold. The ambient temperature was maintained at 10 °C, and each group of experiments was repeated three times, while average values and errors reported.

A cyclic load was applied to the specimen every 1 s, with a loading period of 0.1 s followed by a rest period of 0.9 s. This was repeated 10 times to maintain the horizontal deformation between 150 and 350 microstrain [39]. The resilient modulus was determined using Equation (3).

(3) $$\mathrm{Resilient}\;\mathrm{Modulus}=\frac{P\times G_{L}}{\Delta H\times tDC_{cmpl}}$$

where *P* denotes the peak horizontal load; *G_L_* represents the strain gauge length; *ΔH* signifies horizontal deformation; *t* and *D* correspond to the specimen thickness and diameter; *C_cmpl_* (*C_cmpl_* = 0.6354(*X*/*Y*)^−1^ − 0.332) is the creep compensation factor; and (*X*/*Y*) indicates the ratio of horizontal to vertical deformation.

After obtaining the resilient modulus, the sample was allowed to rest for 15 min. A sustained load was then applied for a duration of 1000 s to achieve a deformation of 150–250 microstrain [39]. Creep compliance was calculated using Equation (4).

(4) $$\mathrm{Creep}\;\mathrm{Compliance}=\frac{\Delta H\times tDC_{cmpl}}{P\times G_{L}}$$

The parameters are the same as those in Equation (3).

Finally, the tensile strength test was carried out by applying a sustained load to the sample at a rate of 0.084 mm/s until fracture. The tensile strength was calculated according to Equation (5).

(5) $$\mathrm{Tensile}\;\mathrm{strength}=\frac{2PC_{sx}}{\pi tD}$$

Stress coefficient C_sx_ = 0.948 − 0.01114*t*/*D* − 0.2693*ν* + 1.436t·*ν*/*D*; Poisson’s ratio *ν* = −0.1 + (1.48 − 0.778*t*^2^/*D*^2^) × (*X*/*Y*)^2^; *P* is the fracture failure load; other parameters are the same as in Equation (3).

As shown in Figure 4b, the fracture energy (FE) can be further divided into elastic energy (EE) and dissipated creep strain energy limit (DCSE). According to the energy relationship, DSCE is calculated using Equation (6).

(6) $$\mathrm{DCSE}=\;\mathrm{FE}-\mathrm{EE}=\int_{0}^{\varepsilon_{f}}\sigma (\varepsilon )d\varepsilon -\frac{1}{2}\sigma_{t}(\varepsilon_{f}-\varepsilon_{0})$$

Strain *ε_0_* is obtained as *ε_0_* = *ε_f_* − *σ_t_/M_R_*.

## 4. Results and Discussion

### 4.1. FTIR Analysis

**Figure 5:**
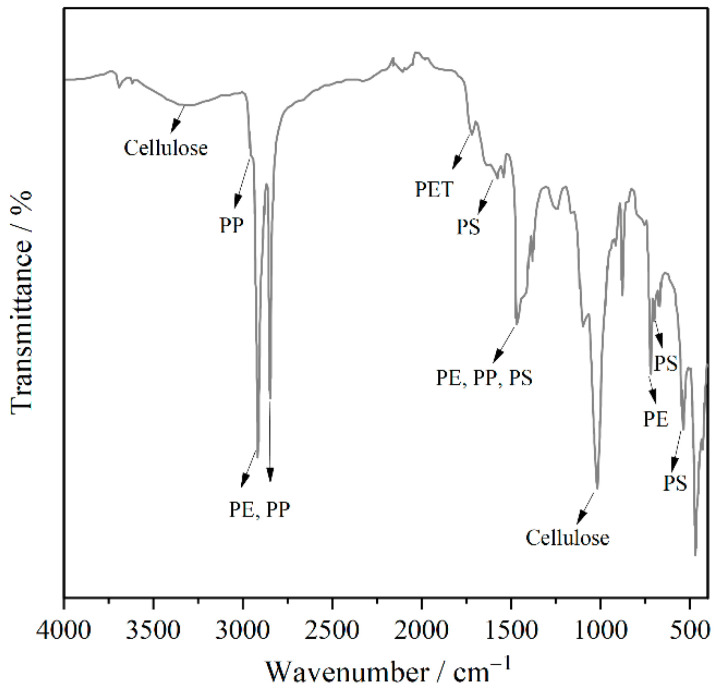
Infrared spectrum of plastic.

As observed in Figure 5, the characteristic absorption bands at 2915 cm^−1^ and 2848 cm^−1^ correspond to the symmetric and asymmetric CH_2_ stretching vibrations of PE. The absorption band observed at 1465 cm^−1^ corresponds to the CH_2_ bending vibration in polyethylene, while the peak identified at 720 cm^−1^ is attributed to the CH_2_ in-plane rocking vibration characteristic of PE. The CH_3_ asymmetric/symmetric stretching vibration (2950–2970 cm^−1^, 2865–2880 cm^−1^) of PP overlaps with PE, the CH_3_ asymmetric bending vibration (1450–1460 cm^−1^) of PP overlaps with PE; and the CH_3_ symmetric bending vibration (1375 cm^−1^) of PP also overlaps with PE.

The spectral region between 2000 cm^−1^ and 1667 cm^−1^ corresponds to out-of-plane C–H bending vibrations of the polystyrene benzene ring, while the absorption peaks in the range of 750–690 cm^−1^ are associated with out-of-plane C–H deformations, both characteristic of a monosubstituted benzene structure. The absorption peak at 1712 cm^−1^ is attributed to the C=O stretching vibration in polyethylene terephthalate (PET). The broad band observed at 3330–3340 cm^−1^ corresponds to O–H stretching vibrations characteristic of cellulose, while the strong absorptions in the 1000–1200 cm^−1^ range are assigned to C–O stretching vibrations of the cellulose backbone.

### 4.2. Thermal Stability

**Figure 6:**
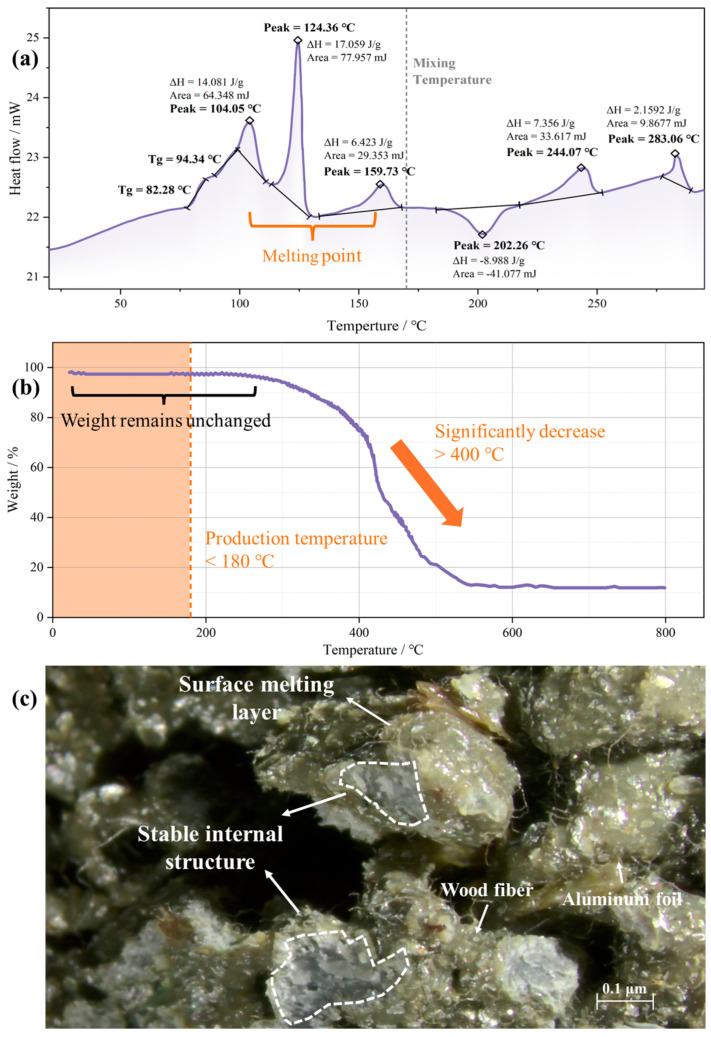
Thermal stability of plastics: (**a**) DSC curve; (**b**) thermogravimetric analysis; (**c**) heating results of recycled plastic.

The DSC test results illustrating the thermal behavior of recycled plastic are shown in Figure 6a. The first melting point, reached at 104 °C, corresponds to LDPE. The subsequent melting points are 124.36 °C (HDPE, LLDPE) and 159.73 °C (PP). The presence of PET (peak = 244.07 °C, 283.06 °C; *T_g_* = 82.28 °C) is detected because the DSC curve is independent of material content, and the identification of trace PET proves the accuracy of the results [40]. The same trace plastic, PS, has no melting point due to its amorphous structure but has *T_g_* = 94.34 °C. Considering that the melting point is below the blending temperature of the mixture, the state of the heated plastic needs to be examined.

As shown in Figure 6b, thermogravimetric analysis revealed that the mass of the plastic did not change within the production temperature range. Significant mass loss only occurs when the temperature exceeds 400 °C. As shown in Figure 6c, after heating at 180 °C (production temperature) for 6 h, only a thin surface layer of the plastic melted, and the internal structure remained very stable. This is because the plastic is a complex multiphase system, and the recycled wood fibers and aluminum foil reinforce its structure [41]. In addition, plastic has very poor thermal conductivity, while aluminum foil reflects heat radiation, insulating the interior and preventing it from absorbing sufficient heat to melt. Therefore, the recycled plastic remains stable during the production process.

### 4.3. Workability

**Figure 7:**
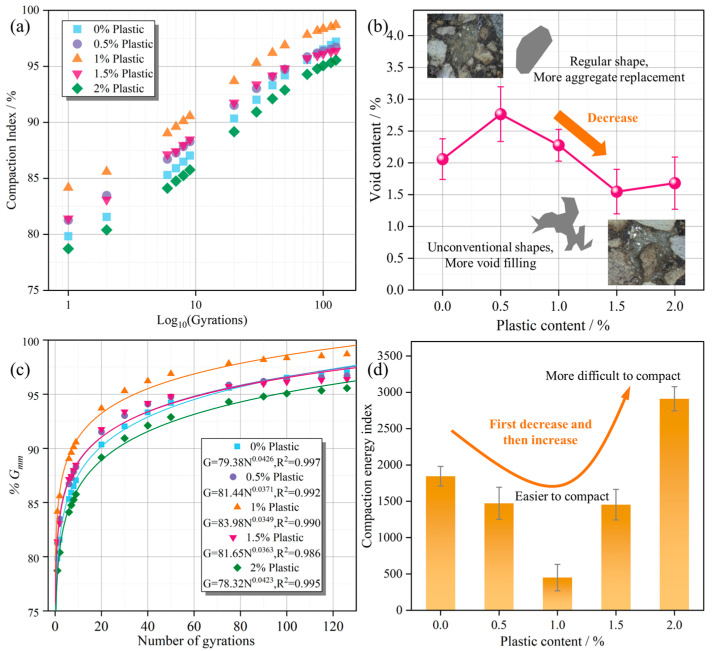
Workability: (**a**) compaction index; (**b**) void content; (**c**) compaction curve; (**d**) compaction energy index.

After confirming the thermal stability of the plastic, its impact on workability was analyzed. As shown in Figure 7a, when the plastic content is lower than 1.5%, the plastic replaces more aggregate, and the compaction index increases because plastic is more easily compacted than aggregate. When the plastic content reaches 2%, the compaction index decreases due to reduced replacement space, as the plastic compresses the original asphalt space.

This can also be observed in the void content (Figure 7b), which decreases with increasing plastic. As the replacement space decreases, the shape of the plastic changes from regular to irregular. The plastic compresses the asphalt and the void spaces, resulting in lower compaction index and void content.

As shown in Figure 7c, the degree of compaction gradually increases with the number of gyrations (*N*), rising rapidly in the initial stage and slowing in the later stage. As shown in Figure 7d, the compaction index first decreases first and then increases. When a small amount of recycled plastic is added, the workability improves, but when the content reaches 2%, it becomes less favorable for construction.

### 4.4. Superpave IDT Evaluation

#### 4.4.1. Resilient Modulus

**Figure 8:**
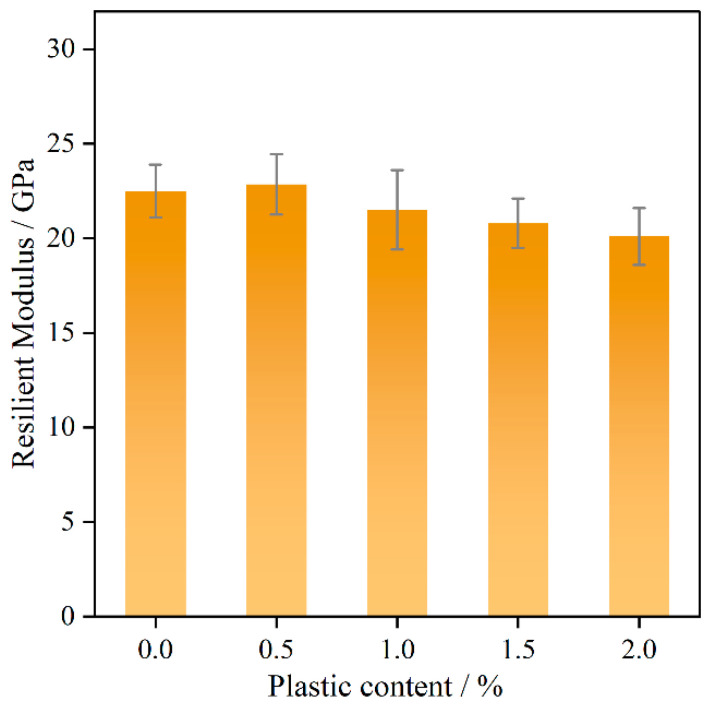
Resilient modulus with different plastic content.

As shown in Figure 8, the resilience modulus of the bitumen mixture fluctuates slightly with the addition of plastics. Considering that the test temperature is much higher than the *T_g_* temperature of PE (−125 °C to −90 °C) and PP (−20 °C to 0 °C), the plastic does not become brittle due to polymer chain segment freezing, this indictes that the resilient modulus is minimally affected within 2% plastic addition. In addition, lower plastic content can result in uneven distribution in the asphalt mixture, leading to insignificant changes in overall performance. During compaction, the plastic changes its morphology, making it less effective than aggregate.

#### 4.4.2. Creep Properties

**Figure 9:**
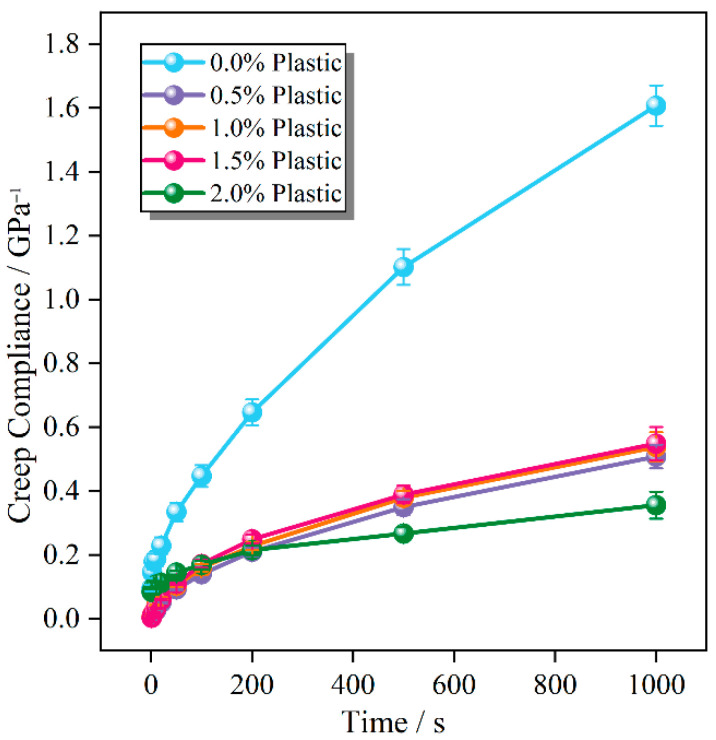
Creep compliance with different plastic content.

As shown in Figure 9, the creep compliance of the asphalt mixture decreases significantly after the addition of plastic, and its resistance to permanent deformation greatly improves [42]. The creep compliance does not differ much when the plastic content ranges from 0.5% to 1.5% and decreases slightly at 2%. The influence of plastic content on creep compliance gradually decreases.

**Figure 10:**
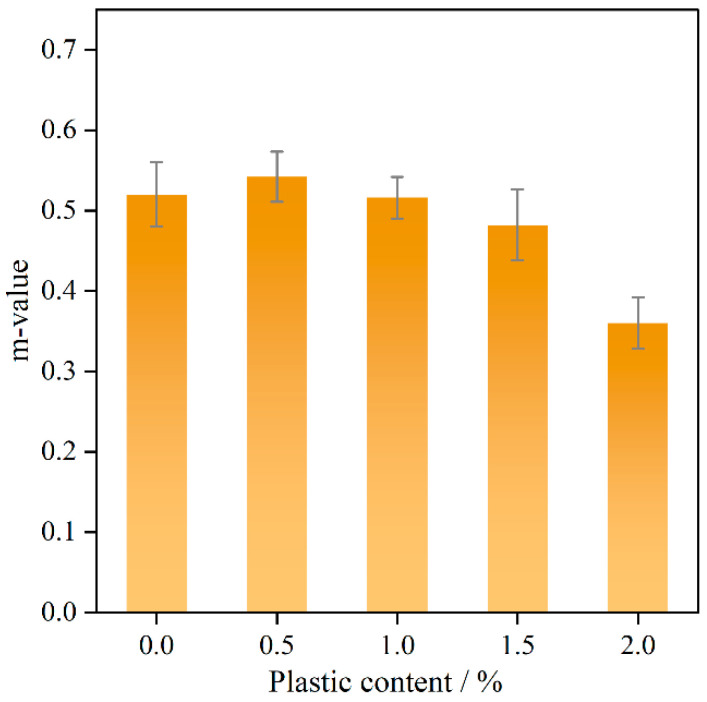
*m*-value with different plastic content.

The *m*-value corresponds to the slope of the creep curve and represents the cumulative damage rate. An increase in the *m*-value indicates higher creep susceptibility, whereas a decrease reflects a reduction in cumulative damage rate [43]. As indicated in Figure 10, at plastic incorporation levels below 1.5%, the *m*-value remainsnearly constant, rising slightly and then decreasing. When the content reaches 2%, the *m*-value decreases significantly due to increased material hardness as the plastic compresses the asphalt and voids.

#### 4.4.3. Tensile Properties

**Figure 11:**
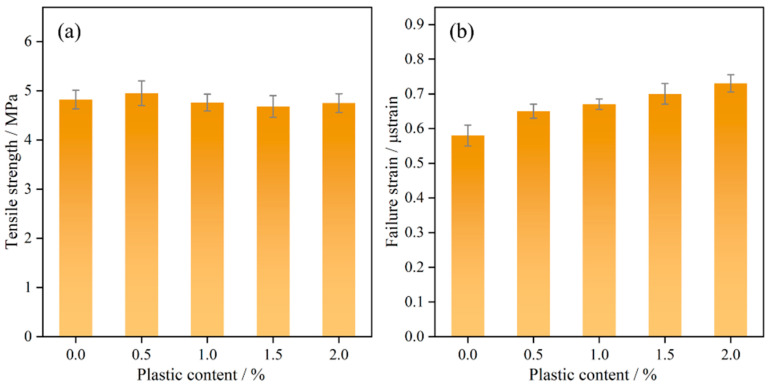
Tensile testing: (**a**) tensile strength; (**b**) failure strain.

The tensile strength, depicted in Figure 11a, exhibits minimal variation with increasing plastic content, showing a reduction of less than 5.6%. Incorporating up to 2% plastic content has negligible impact on tensile strength. Figure 11b shows that the tensile failure strain continues to increase with higher plastic content because the plastic tends to form fibers during the tensile process, which enhances the toughness of the material [44]. The improvement in tensile behavior mainly stems from the ductility of the plastic.

#### 4.4.4. Fracture Energy

**Figure 12:**
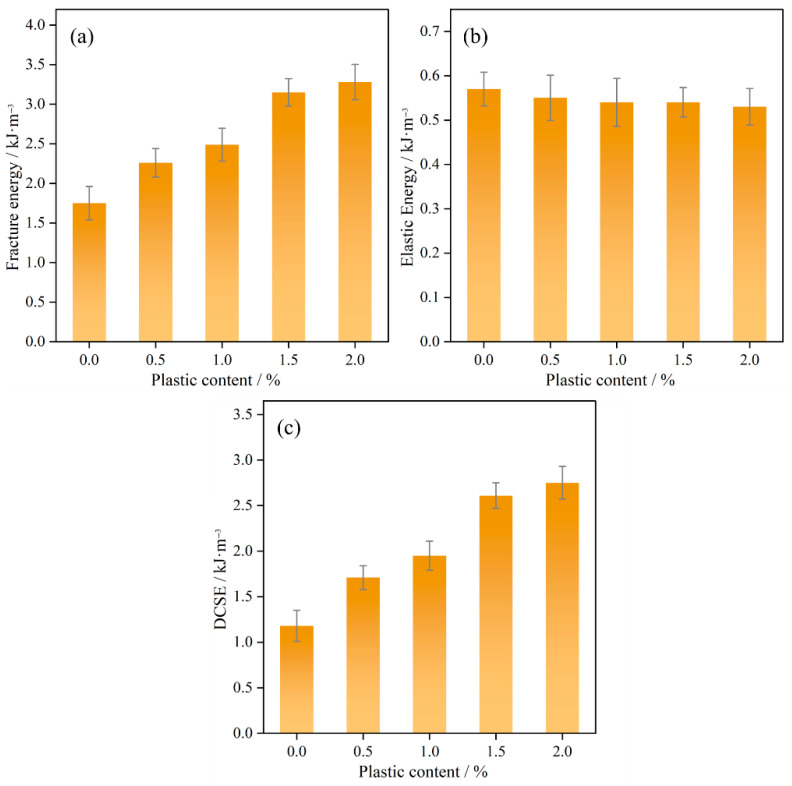
Fracture energy: (**a**) fracture energy; (**b**) elastic energy; (**c**) DCSE.

As illustrated in Figure 12a, the fracture energy of bituminous mixtures increases continuously with higher plastic content. According to Figure 12b,c, this observed rise in fracture energy primarily results from an increase in DCSE. The elastic energy decreased by no more than 7%, while DSCE increased by up to 133.05%. The material toughness is significantly enhanced, and the recycled plastic increases the dissipated energy during crack propagation.

### 4.5. Fracture Mechanism of Plastic Asphalt Mixture

**Figure 13:**
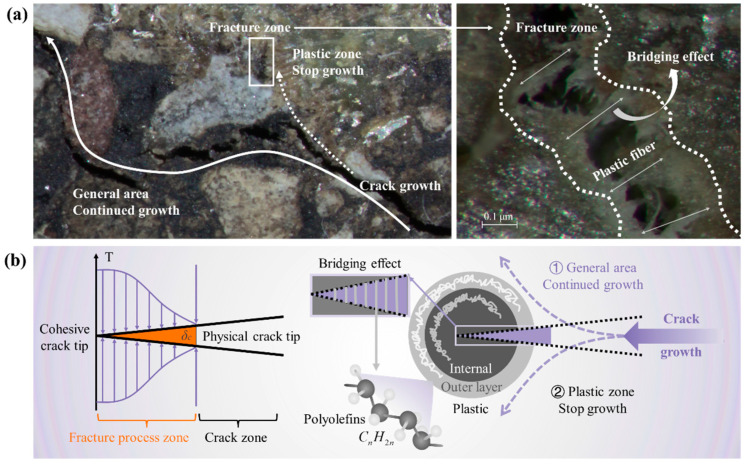
Fracture of asphalt mixture containing plastic: (**a**) fracture diagram; (**b**) fracture theory.

The fracture mechanism of plastic asphalt mixtures is summarized in Figure 13. As shown in Figure 13a, cracks tend to propagate in general areas, while an obvious bridging effect occurs in the fracture zone. Since the recycled plastic is composed of polyolefins (LDPE, LLDPE, HDPE, PP), both the testing and application temperatures are much higher than *Tg*, providing the material excellent toughness and ductility.

As shown in Figure 13b, the nonlinear fracture process is described by the cohesive zone model, which is mainly divided into four stages: (1) no damage; (2) crack initiation; (3) material softening; and (4) critical crack [45]. The crack propagation path mainly occurs in the general area and the plastic zone. Due to heating during production, the plastic forms a double-layer structure. When cracks grow in the plastic zone, the polyolefin molecular chains rearrange and slip to form plastic fibers that hinder propagation. Based on Griffith’s theory, cracks tend to follow the path of least local energy, bypassing the hard second-phase particles [46]. Plastic reduces fracture initiation and alters the crack propagation path, resulting in fewer cracks than in general areas.

## 5. Conclusions

This work aimed to evaluate the feasibility of using recycled plastics from food packages in asphalt pavements. A series of experimental procedures were performed to assess the thermal stability, workability, and cracking performance of the modified asphalt mixture using recycled plastics based on Superpave IDT. The results show that incorporating recycled composite plastics into roads is feasible. The core conclusions are summarized as follows:

1. The recycled plastic used for food packaging is a polyolefin composite plastic primarily composed of LDPE, LLDPE, HDPE, and PP. The wood cellulose and aluminum foil contained in the material improve its thermal stability, enabling the plastic to withstand actual production temperatures.
2. When less than 2% plastic (by aggregate weight) is added, the asphalt mixture exhibits good workability. As the plastic content increases, the plastics begin to occupy spaces within the asphalt and voids, and their role gradually shifts from aggregate replacement to void filling. Meanwhile, the shape of the plastic changes from regular to irregular.
3. When the plastic content increased from 0.5% to 2.0%, creep compliance decreased from 68.4% to 77.87%, while the *m*-value, tensile strength, and maximum elastic energy decreased by 30.77%, 5.6%, and 7%, respectively. The ability of the recycled plastic–asphalt mixture to resist permanent deformation is improved.
4. In contrast, the failure strain, fracture energy, and maximum DSCE increased by 25.86%, 87.43%, and 133.05%, respectively. Adding up to 2% plastic (by aggregate weight) improves toughness and cracking resistance, increasing dissipated energy during crack propagation.
5. When cracks propagate into the plastic zone, the polyolefin molecular chains rearrange and slip, forming plastic fibers that hinder crack extension through a bridging effect. As a result, cracks tend to bypass the plastic zone, and the plastic zone exhibits fewer cracks than the surrounding regions.

## 6. Future Work

This paper focuses on the workability and crack resistance of recycled plastics from food packaging. In the future, the application and overall performance of high-dosage plastics should be further studied. Multiple repeated experiments and statistical analysis methods will be employed to verify the reliability of recycled plastic roads.

## Data Availability

All data are contained within the article.
